# Supplementary figures and images for: Sex hormone-binding globulin, testosterone and type 2 diabetes risk in middle-aged African women: exploring the impact of HIV and menopause
Source: medRxiv. 2024 Dec 29:2024.12.25.24319619. Preprint. [Version 1] doi: 10.1101/2024.12.25.24319619 (PMC11703316; doi:10.1101/2024.12.25.24319619)

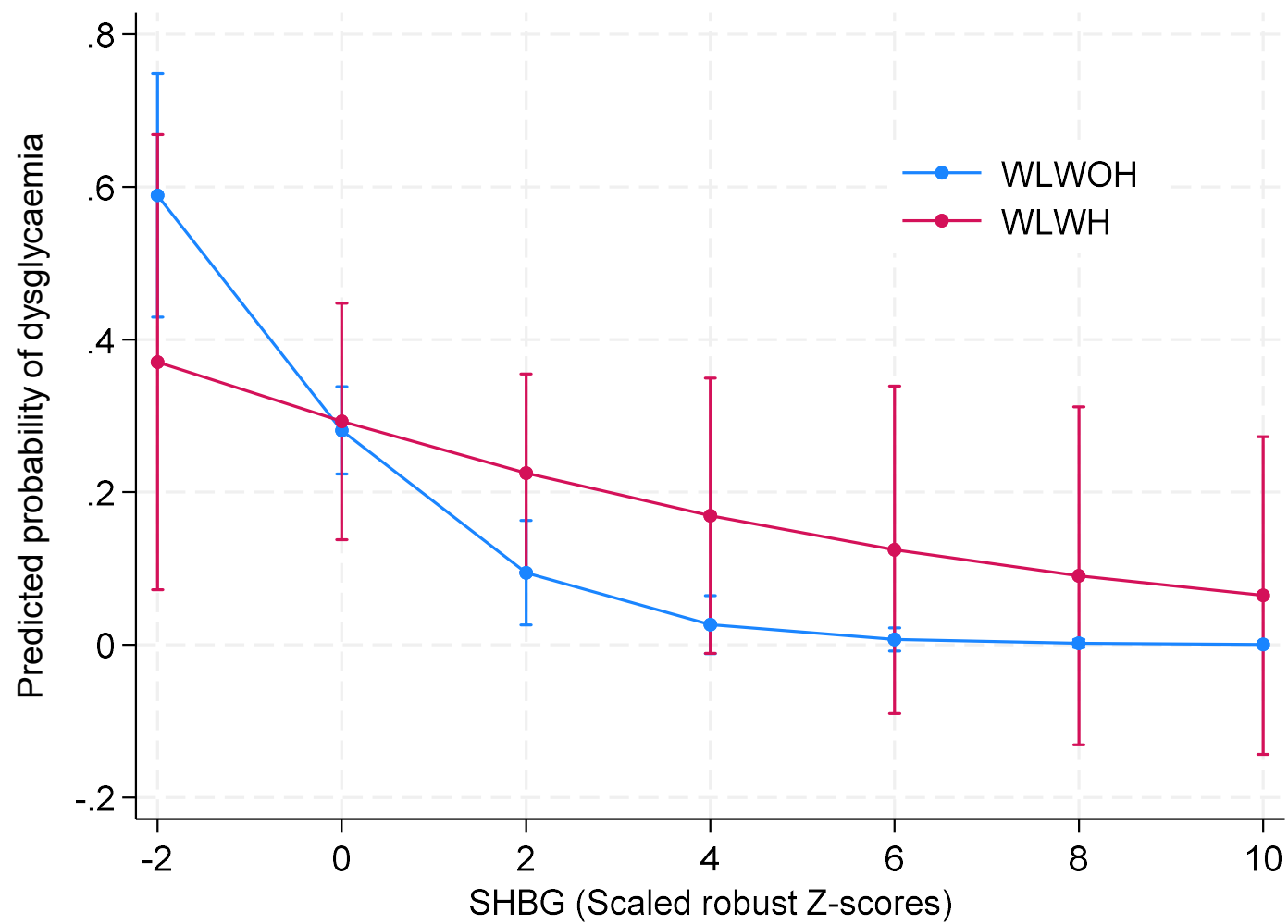

Supplement: Supplement 1 [file media-1.pdf]

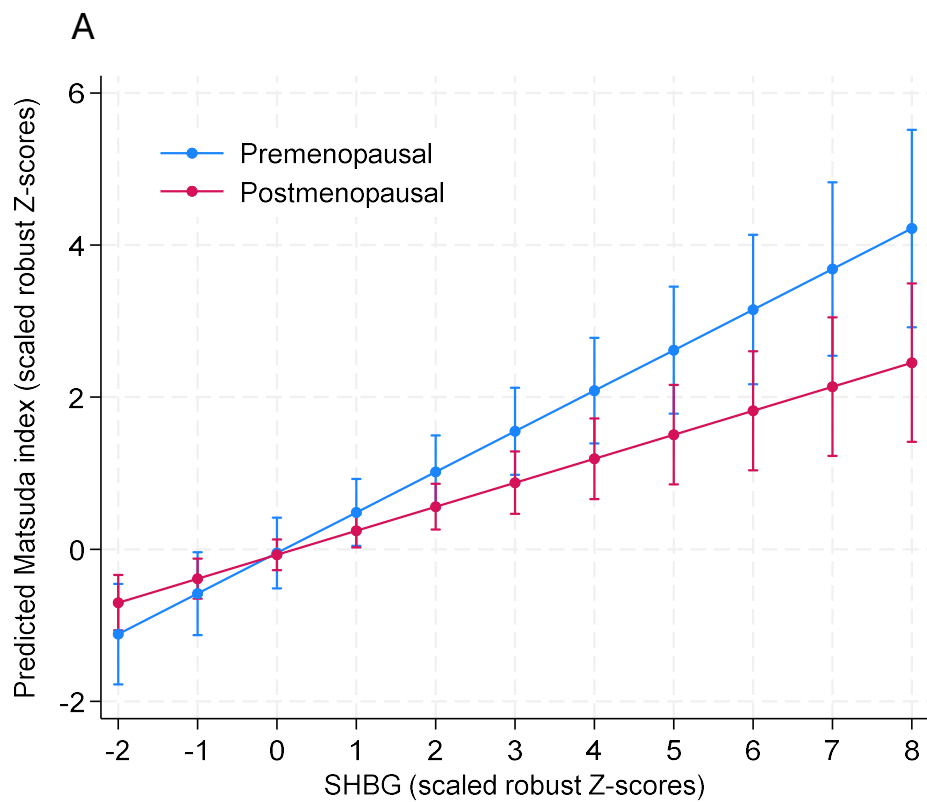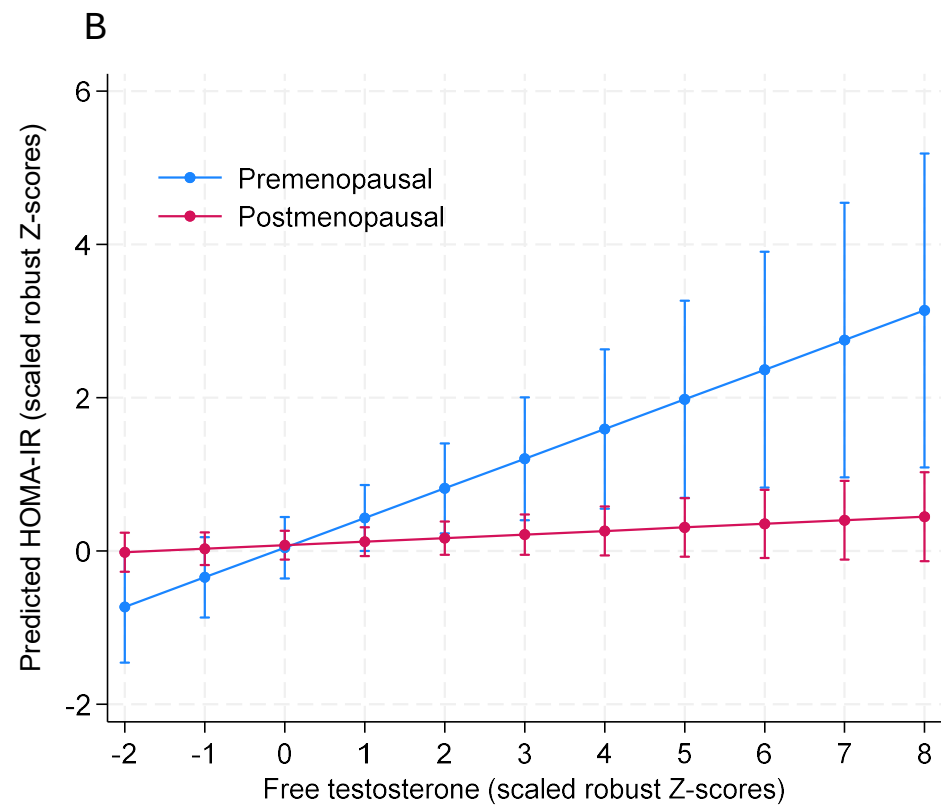

Supplement: Supplement 2 [file media-2.pdf]
